# Plasma Hydrogen Sulfide Is Positively Associated With Post-operative Survival in Patients Undergoing Surgical Revascularization

**DOI:** 10.3389/fcvm.2021.750926

**Published:** 2021-10-25

**Authors:** Alban Longchamp, Michael R. MacArthur, Kaspar Trocha, Janine Ganahl, Charlotte G. Mann, Peter Kip, William W. King, Gaurav Sharma, Ming Tao, Sarah J. Mitchell, Tamás Ditrói, Jie Yang, Péter Nagy, C. Keith Ozaki, Christopher Hine, James R. Mitchell

**Affiliations:** ^1^Department of Vascular Surgery, Centre Hospitalier Universitaire Vaudois and University of Lausanne, Lausanne, Switzerland; ^2^Department of Biomedical Sciences, University of Lausanne, Lausanne, Switzerland; ^3^Department of Health Sciences and Technology, Eidgenössische Technische Hochschule (ETH) Zürich, Zurich, Switzerland; ^4^Department of Surgery and the Heart and Vascular Center, Brigham and Women's Hospital and Harvard Medical School, Boston, MA, United States; ^5^Department of Molecular Metabolism, Harvard T. H. Chan School of Public Health, Boston, MA, United States; ^6^Department of Molecular Immunology and Toxicology, National Institute of Oncology, Budapest, Hungary; ^7^Department of Cardiovascular and Metabolic Sciences, Cleveland Clinic Lerner Research Institute, Cleveland, OH, United States; ^8^Department of Anatomy and Histology, University of Veterinary Medicine, Budapest, Hungary

**Keywords:** hydrogen sulfide, peripheral artery disease (PAD), limb ischemia, biomarker, surgery

## Abstract

**Objective:** Hydrogen sulfide (H_2_S) is a gaseous signaling molecule and redox factor important for cardiovascular function. Deficiencies in its production or bioavailability are implicated in atherosclerotic disease. However, it is unknown if circulating H_2_S levels differ between vasculopaths and healthy individuals, and if so, whether H_2_S measurements can be used to predict surgical outcomes. Here, we examined: (1) Plasma H_2_S levels in patients undergoing vascular surgery and compared these to healthy controls, and (2) the association between H_2_S levels and mortality in a cohort of patients undergoing surgical revascularization.

**Methods:** One hundred and fifteen patients undergoing carotid endarterectomy, open lower extremity revascularization or lower leg amputation were enrolled at a single institution. Peripheral blood was also collected from a matched control cohort of 20 patients without peripheral or coronary artery disease. Plasma H_2_S production capacity and sulfide concentration were measured using the lead acetate and monobromobimane methods, respectively.

**Results:** Plasma H_2_S production capacity and plasma sulfide concentrations were reduced in patients with PAD (*p* < 0.001, *p* = 0.013, respectively). Patients that underwent surgical revascularization were divided into high vs. low H_2_S production capacity groups by median split. Patients in the low H_2_S production group had increased probability of mortality (*p* = 0.003). This association was robust to correction for potentially confounding variables using Cox proportional hazard models.

**Conclusion:** Circulating H_2_S levels were lower in patients with atherosclerotic disease. Patients undergoing surgical revascularization with lower H_2_S production capacity, but not sulfide concentrations, had increased probability of mortality within 36 months post-surgery. This work provides insight on the role H_2_S plays as a diagnostic and potential therapeutic for cardiovascular disease.

## Introduction

Hydrogen sulfide (H_2_S) is a redox modifying and diffusible gasotransmitter that plays numerous physiologic roles across various organ systems including the cardiovascular system (1). While high levels of exogenous H_2_S are toxic, increased levels within a supraphysiologic range have been shown to mediate many beneficial effects ranging from stress resistance to longevity (2–4). Exogenous H_2_S has been shown to extend lifespan and increase stress resistance in *Caenorhabditis elegans (C. elegans)* and *Drosophila melanogaster (D. melanogaster)*, and to protect mice from lethal levels of hypoxia, although effects on human lifespan and survival remain to be determined (2, 5). H_2_S has also emerged as a critical mediator of vascular homeostasis (6) in preclinical models through its functions as a vasodilator (7), antioxidant (8), oxygen sensor (9, 10), immunomodulator (11), and anti-inflammatory gas (12). Individuals with diabetes-related vascular inflammation had decreased circulating sulfide levels (13). As atherosclerosis is a chronic inflammatory disease, it is important to note H_2_S is shown to regulate several atherosclerotic cellular and inflammatory processes (14–17).

While H_2_S has been implicated in the pathogenesis of multiple cardiovascular disease processes, the measurement of H_2_S is arduous and often relies on indirect measures and surrogates (18–23). Nevertheless, further characterizing the role of H_2_S in systemic disease states such as peripheral arterial disease (PAD) may yield important insights on potential diagnostic and therapeutic applications.

Thus, our objective was to assess the clinical relevance of plasma H_2_S levels in patients suffering from vascular disease. Individuals included in this study were diagnosed with either carotid artery stenosis requiring carotid endarterectomy (CEA), or peripheral artery disease (PAD) necessitating revascularization or amputation secondary to unsalvageable critical limb ischemia. Patients were followed for 36 months post-surgery with clinical outcomes and mortality measured. A baseline control cohort of patients matched for age, sex, and hypertension (7), but with no history of PAD, prior MI, coronary interventions, heart failure, or stroke were included for comparing H_2_S levels in the diseased vs. healthy state. Our study provides new insight into the relevance of circulating H_2_S levels in patients suffering from atherosclerosis, and during surgical revascularization.

## Materials and Methods

### Study Design and Demographic Data Collection

The study followed the principles of the Declaration of Helsinki and was approved by the Partners Human Research Committee institutional review board (IRB). Informed consent was obtained for prospective collection of demographic and clinical data. All consecutive patients, at a single institution undergoing elective vascular surgery from 2012 to 2015, scheduled for carotid endarterectomy, open lower extremity revascularization, and major lower extremity amputation (secondary to critical limb ischemia) were enrolled. None of the amputations were traumatic. Patients were excluded if they were <18 years, had an emergent indication for the operation or if they were involved in another clinical research study. The cohort baseline risk factors and demographics were collected including age, race, history of PAD, history of stroke or myocardial infarction as well as history of coronary intervention (Supplemental Table 1). To compare levels of H_2_S between patients with vascular disease vs. healthy patients, an additional group of 20 control subjects were enrolled without documented or diagnosed PAD or CAD. This control cohort was matched for age, sex and hypertension, and was randomly extracted from the Brigham and Women's biobank. No additional clinical information was provided for this control cohort.

### Hydrogen Sulfide Measurements

On the day of surgery, but prior to surgical intervention, peripheral venous blood was collected into ethylenediaminetetraacetic acid (EDTA) collection tubes at the time of peripheral intravenous line placement. The tube was inverted several times to ensure mixing with the anticoagulant and then transferred to the lab for processing. To obtain plasma all samples were centrifuged at room temperature for 15 min at 2,000 g, plasma was then stored at −80°C for future analysis. All samples were then thawed and processed for H_2_S production capacity at once to ensure uniformity.

Hydrogen sulfide production capacity was measured using the lead acetate method (24). In brief, plasma was mixed with 150 μl freshly prepared reaction mixture, containing 100 mM L-cysteine and 0.5 mM pyridoxal 5'-phosphate (PLP, aka Vitamin B6) in Phosphate buffered saline (PBS) in a plastic 96-well plate. The plate was then incubated at 37°C with lead acetate embedded filter paper on top. Upon the reaction of H_2_S with the lead acetate paper, a dark lead sulfide precipitate is produced. The paper was incubated for 6 h, until a detectable, but non-saturated signal was seen.

*In vitro* H_2_S production assays to demonstrate sensitivity and validity of the lead acetate assay to detect iron-catalyzed H_2_S production were similarly performed as previously described (25). Briefly, 150 μL reactions in PBS were set up in 96-well plates. Each reaction contained 10 mM L-cysteine, and according to the respective conditions as indicated in the figures, 0–50 μM FeCl_3_, 0–50 μM hemin, 0–1,000 μM PLP, and/or 0 to 50 mM homocysteine. The lead acetate filter paper was then placed on top of the reaction plates with a weight on top and incubated at 37°C for 1.5–2 h.

The amount of lead sulfide captured on the paper was quantitated by using the IntDen measurement function in ImageJ software and normalized to the respective control group after subtracting the background. An empty well without plasma, was used as blank value was used for background measurement. These measurements were assessed by 2 independent investigators blinded to group assignment.

Hydrogen sulfide concentration determination using the MBB method was conducted as described previously (26). Briefly: In almost complete darkness due to the light sensitivity of the MBB reagent, 20 μl of 50 mM HEPES (pH = 8.0) buffer and 20 μl of 10 mM monobromo-bimane (Sigma Aldrich) was added to 20 μl of plasma sample. After 10 min in the dark at room temperature, the product sulfide-dibimane was extracted with 200 μl of pure ethyl acetate. The organic supernatant was collected and evaporated to dryness and stored at −20°C until measurement. The solid samples were redissolved prior to HPLC measurements in acetonitrile. Ten microliter was injected and separated on an Agilent Eclipse XDB-C18 (4.6 × 250 mm, 5 μm) using a Merck Hitachi L7000 HPLC instrument with a Thermo UltiMate 3000 fluorescent detector. The elution method employed a 28 min long gradient using water and acetonitrile both containing 0.1% TFA. The detection of the product was carried out using UV-absorbance measurement at 254 nm and fluorescent measurement with extinction at 390 nm and detection at 480 nm. Quantitation was done using a standard calibration curve in aqueous buffered solutions, where H_2_S concentrations were verified by the DTNB assays. It should be noted that the MBB method measures endogenous sulfide levels that are easily liberated from the bound plasma sulfide pool and hence absolute values largely depend on the applied conditions (temperature, alkylation time, concentration conditions etc). This is the reason why absolute sulfide values obtained here should not be compared with values reported in other studies (23).

The enzymatic activity of CBS in blood samples was measured by an HPLC-MS/MS protocol previously published (27). Briefly, 20 μL of plasma sample was added to 25 μL of solution containing 200 mmol/L Tris-HCl (pH 8.6), 1 mmol/L pyridoxal 5′-phosphate, 1 mmol/L SAM and 40 mmol/L 2,3,3-2H-labeled serine (Cambridge Isotope Laboratories, Inc). Five microliter of starting solution containing 280 mmol/L homocysteine thiolactone in 100 mmol/L Tris-HCl (pH 8.6), 10 mmol/L DTT, 1.225 mol/L NaOH was incubated for 5 min at 37°C to produce homocysteine by thiolactone cleavage, pH was adjusted to 8.6 with 1:1 HCl solution and the solution was added to the plasma mixture. After 4 h of incubation at 37°C, the reaction was quenched by acidification of the mixture with 100 μL of EZ:faast Internal Standard Solution (Phenomenex) containing 3.3 μmol/L of internal standard 3,3,4,4-2H-labeled cystathionine. Sample preparation was carried out using EZ:faast kit (Phenomenex) kit, that included a solid phase extraction step, derivatization with propyl chloroformate, and an extraction into an organic solvent. The prepared samples were separated on an EZ:faast AAA-MS column (250 × 2.0 mm, Phenomenex) using LC and MS settings described in the EZ:faast user manual using a Thermo Scientific UltiMate 3000 HPLC connected to a Thermo Scientific LTQ-XL MS instrument. Concentrations (and enzyme activities) were calculated using the internal standard.

### Statistical Analysis

Plasma H_2_S production capacity and sulfide concentration in vascular surgery patients and healthy controls were compared using Student's *t*-test. Pearson correlation coefficient was calculated to assess the association between plasma H_2_S production capacity and plasma sulfide concentration. High low H_2_S production capacity/free sulfide groups and groups were divided by median split, which was arbitrary. The Kaplan-Meier method was used to estimate survival in high and low H_2_S production capacity/free sulfide groups and groups were compared using log-rank test. Cox proportional hazard models were fit to estimate mortality hazard ratios between high and low H_2_S producing groups, corrected for potentially confounding variables (age, gender, BMI). Both unadjusted and adjusted Cox proportional hazard models met assumptions of proportional hazard. To assess the odds of post-surgical complication in high vs. low H_2_S producing individuals, Fisher's exact test was used. Statistical tests were performed using Graphpad Prism 7 and R version 3.3.2. Kaplan-Meier curves and Cox proportional hazard models were fit in R using the Survival package and Kaplan-Meier curves were visualized using the Survminer package. All reported *P* values are based on 2-sided tests and *P* values of <0.05 were considered statistically significant.

## Results

### H_2_S Levels Are Reduced in Patients With Vascular Disease Compared to Patients With No Documented Atherosclerosis

The characteristics of the control and the vascular cohort are summarized in Table 1. Mean (SD) age of the control and vascular patients was 68 (2.3) and 69 (9), respectively. These patients only differed in the prevalence of PAD, prior MI/coronary revascularization, and hyperlipidemia (Table 1). The control cohort included a group of 20 subjects, matched for age, sex and hypertension, but with no documented atherosclerotic cardiovascular disease. The vascular cohort included a heterogenous group of 115 patients. Forty nine underwent carotid endarterectomy (asymptomatic *n* = 34, symptomatic *n* = 15), 44 infra-inguinal revascularization (claudication *n* = 21, resting pain *n* = 15, tissue loss *n* = 13), and 22 amputation.

**Table 1:** Baseline study population characteristics.

**Variable**	**Control cohort**	**Vascular cohort**	***P***
	***n* = 20**	***n* = 115**	
Age (SD)	68 (2.3)	69 (9)	0.62
Male (%)	13 (65)	73 (63)	0.89
Ethnicity (%)			
White	18 (90)	97 (84)	0.51
Hispanic	0	10 (9)	
Black	0	4 (3.5)	
Other	2 (10)	4 (3.5)	
BMI (SD)	28.7 (7.2)	28.3 (5.3)	0.76
Comorbidities (%)			
Heart failure	0	19 (17)	0.16
History of stroke	0	24 (21)	0.08
PAD	0	75 (65)	<0.01
Prior coronary intervention	0	44 (38)	<0.01
Prior MI	0	30 (26)	0.03
Hypertension	19 (95)	106 (92)	0.65
Hyperlipidemia	6 (30)	98 (85)	<0.01
COPD	0	7 (6)	0.81
Renal dysfunction	1 (5)	16 (14)	0.24
Diabetes mellitus	2 (10)	45 (39)	0.01
Smoking status (%)			
Never	9 (45)	25 (22)	0.03
Former	11 (55)	60 (52)	0.81
Current	0	30 (26)	0.03
Procedure type (%)			
Carotid endarterectomy	0	49 (43)	
Asymptomatic	0	34 (69)	
Symtomatic	0	15 (31)	
Infra-inguinal revascularization	0	44 (38)	
Claudication	0	21 (48)	
Resting pain	0	15 (34)	
Tissue loss	0	13 (30)	
Lower extremity amputation	0	22 (19)	
Medications			
Aspirin		102 (89)	
ACE inhibitor		49 (43)	
Beta blocker		89 (78)	
Coumadin		11 (10)	
Ca^2+^ channel blocker		37 (32)	
Statin		99 (86)	
Fibrate		3 (3)	
Metformin		8 (7)	

*SD, standard deviation; BMI, body mass index; MI, myocardial infarct; HTN, hypertension; COPD, chronic obstructive pulmonary disease*.

**Figure 1:**
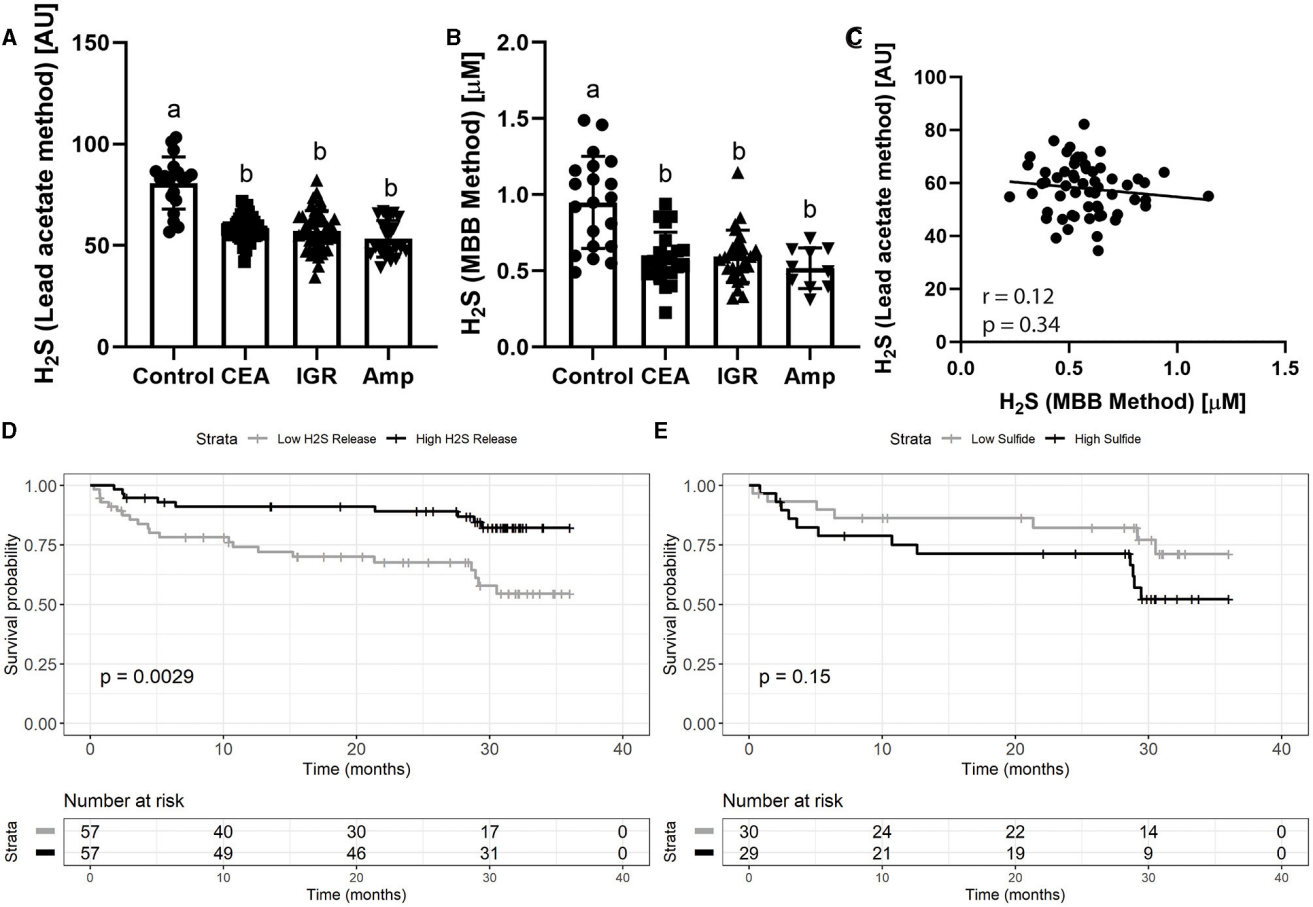
Plasma H_2_S production capacity and sulfide levels are reduced in patients with vascular disease, with production capacity predicting mortality. **(A)** Plasma H2S production capacity and **(B)** plasma sulfide measured by the MBB method from human patients suffering vascular occlusive disease including 115 patients undergoing carotid endarterectomy (CEA), infra-inguinal revascularization (IGR) and amputation (Amp), as well as age-matched individuals (*n* = 20). Error bars indicate SD; different letters indicate difference *p* < 0.05 in One Way ANOVA Tukey *post-hoc* test. **(C)** Correlation between H_2_S production capacity and sulfide measured by the MBB method in vascular disease patients. **(D)** Probability of survival for vascular disease patients during follow up after intervention with low (*n* = 57) vs. high (*n* = 57) H_2_S production capacity and **(E)** low and high plasma sulfide measurements. High vs. low was determined by median split. P value calculated from log-rank test.

Analysis of the baseline blood sample (pre-operative) H_2_S production capacity assay using the lead acetate-based method, showed that controls had significantly higher H_2_S production capacity compared to vascular disease patients (Figure 1A; Supplementary Figure 1A; 80.8±12.9 vs. 57.0 ± 8.4 arbitrary units, *p* < 0.001). Importantly, H_2_S levels was similarly reduced in patient that underwent carotid endarterectomy (CEA, 58.6 ± 5.79), infra-inguinal revascularization (IGR, 57.1 ± 10.1) and amputation (Amp, 53.3 ± 8.97). Results using the MBB method also showed that healthy controls had significantly greater plasma sulfide levels (Figure 1B; 0.95 ± 0.30 vs. 0.58 ± 0.16 μM, *p* < 0.05), and was consistent across the underlying vascular surgeries (CEA 0.59 ± 0.17, IGR 0.59 ± 0.17, Amp 0.52 ±0.13; Figure 1B). Although both of these sulfide-based measurements were significantly higher in healthy controls, there was no observable correlation between the measurements (Figure 1C; *r*_s_ = −0.12, *p* = 0.34), suggesting that the two methods measure fundamentally different phenomena and/or sulfide pools.

### H_2_S Levels Correlate With Post-operative Survival

To assess the associations of these H_2_S measurements with clinical outcomes after surgical procedures, the vascular surgery patients were divided by median split into patients with high and low pre-operative H_2_S production capacity (Supplementary Figure 1B). Over 36 months of follow up, patients with low H_2_S production capacity had significantly decreased probability of survival compared to patients with high H_2_S production capacity (Figure 1D; 54 vs. 82% survival probability, log-rank test *p* = 0.0029). However, when patients were divided by median split into high and low MBB-measured sulfide measurements, there was no significant association with survival and the trend was reversed such that those with higher sulfide tended to have lower probability of survival (Figure 1E; 71 vs. 52% survival probability, log-rank test = 0.15).

**Figure 2:**
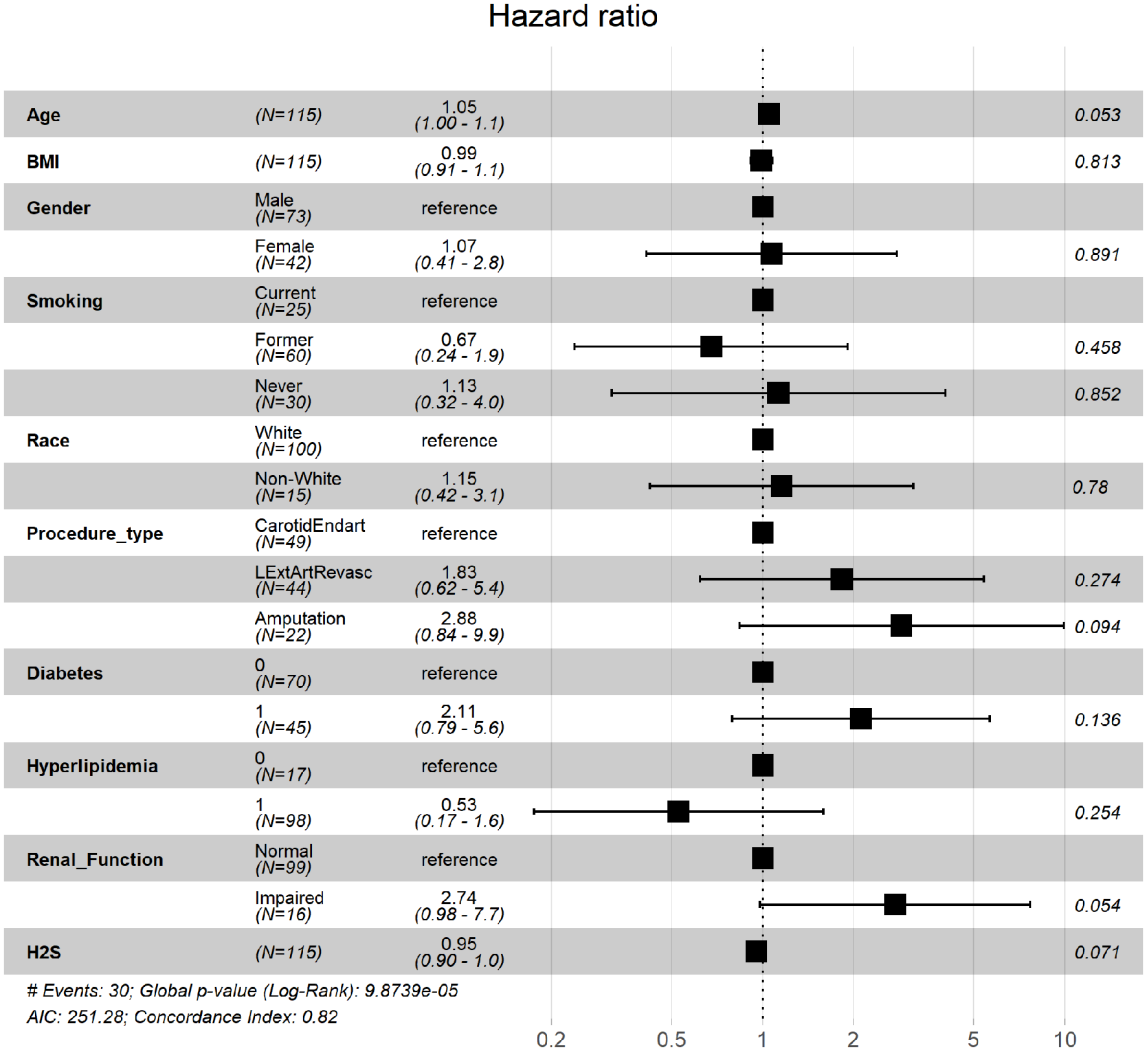
Hazard ratios of death during follow up period. Hazard ratio estimates and 95% confidence intervals for clinically relevant parameters and plasma H_2_S production capacity as measured by the lead acetate release method.

To further assess the robustness of H_2_S production capacity as a predictor of mortality after a vascular surgery intervention, we generated Cox proportional hazard models to adjust for potential confounding variables. A univariate unadjusted model showed that individuals in the high H_2_S production capacity group had significantly reduced risk of death during follow up (HR(95% CI) = 0.31(0.11–0.87), coefficient *p* = 0.025, model Wald *p* = 0.025). In a Cox proportional hazard model adjusted for age, BMI, gender, smoking history, race, procedure type, diabetes, hyperlipidemia, and renal function, H_2_S production capacity was significantly associated with survival (HR = 0.95, CI = 0.90–1, coefficient *p* = 0.07, model Wald *p* < 0.001; Figure 2).

### H_2_S Production Reflects a Non-enzymatic Process

**Figure 3:**
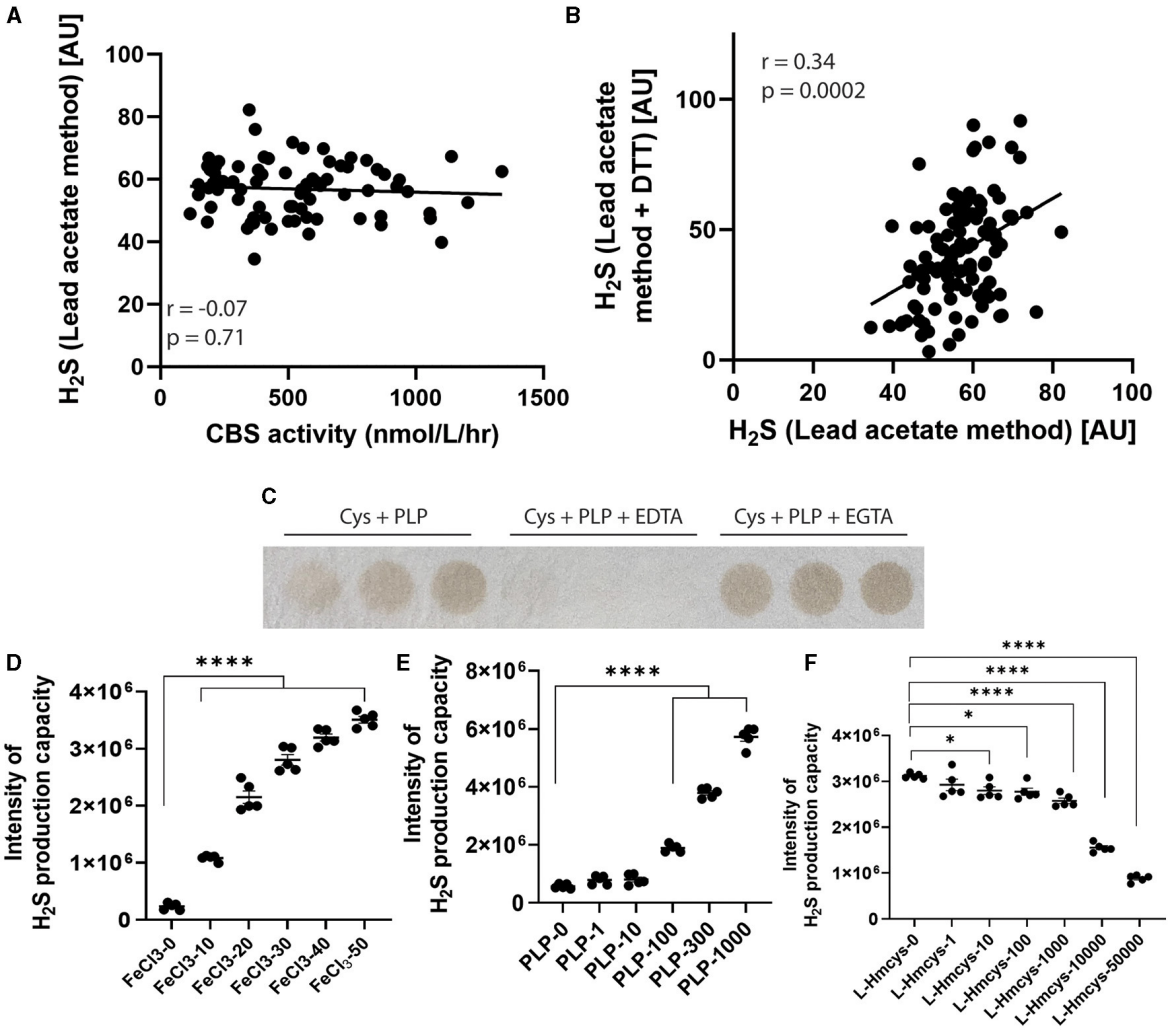
Mechanisms of H_2_S release from plasma. **(A)** Correlation between plasma cystathionine-beta synthase activity and plasma H_2_S release. **(B)** Correlation between plasma H_2_S release performed under protein non-denaturing (x-axis) and protein denaturing (y-axis) conditions. **(C)** H_2_S release from plasma when incubated with PLP, PLP + EDTA or PLP + EGTA. **(D)** *In vitro* H_2_S release performed using cysteine and increasing concentration of FeCl_3_, **(E)** PLP or **(F)** homocysteine (*n* = 5 reactions per condition). *p < 0.05, ****p < 0.0001 vs. respective 0uM control.

Finally, to characterize the biochemical nature of the plasma H_2_S production capacity measurement, we investigated whether it might be generated by enzymatic or non-enzymatic production. It has been proposed that the H_2_S generating enzyme cystathionine beta synthase (CBS) is present in human plasma. We measured plasma CBS activities, but found no observable association between plasma CBS activity and H_2_S production capacity (Figure 3A; *r* = −0.07, *p* = 0.71). We next performed the assay under protein denaturing conditions by heating and adding DTT, and found a highly correlated pattern of signals compared to when the assay was performed under non-denaturing conditions (Figure 3B; *r* = 0.34, *p* = 0.0002). These results suggest a non-enzymatic process for H_2_S production in the plasma. Finally, we added chelators and found that EDTA was able to almost completely ablate the H_2_S release, while EGTA was not (Figure 3C). On the other hand, EDTA had a reverse effect on measured sulfide concentrations by the MBB method (23) corroborating that the two methods measure sulfide released from fundamentally different endogenous pools and/or production mechanisms. This suggests a vital role for free and bound iron as a catalyst in H_2_S release. In combination with PLP and the substrate L-cysteine, H_2_S release is ultimately dampened by increasing homocysteine concentrations (Figures 3D–F; Supplementary Figures 1C–E). These results on the likely non-enzymatic nature of the signal are in agreement with the recent thorough characterization of mechanisms of H_2_S release in blood by Hine et al. (25).

## Discussion

Pre-clinical studies have suggested that decreased levels of H_2_S accelerate the development of atherosclerosis (14), and are reduced in the skeletal muscle of CLI patients (1). Here we show that patients with vascular disease have significantly decreased circulating H_2_S production capacity and sulfide concentrations, compared to subjects with no clinical evidence of coronary or peripheral artery disease. Together, these results suggest that patients with atherosclerotic vascular disease have a decreased capability to generate H_2_S. Furthermore, patients with higher H_2_S production capacity measured prior to vascular surgery had reduced post-operative mortality at 36 months follow-up compared to those with lower H_2_S production. It also suggests possible therapeutic application of H_2_S donors, to restore or increase H_2_S levels in humans. Indeed, the administration of exogenous H_2_S (NaHS, SG1002) protected against ischemia-induced heart failure, and improved overall survival in rodents (28, 29). Such an H_2_S donor is being tested in clinical trials (NCT01989208). Similarly, exogenous administration of H_2_S in preclinical models protects from ischemic injury to the liver (2), the brain (30), and the kidney (31), which could have therapeutic benefits in the context or solid organ transplantation, myocardial infarction or stroke. Interestingly, some FDA approved drug, with H_2_S-releasring properties, such as the ACE inhibitor Zofenopril or sodium thiosulfate, improve vascular function, and limit intimal hyperplasia (unpublished data).

Interestingly, this association was not observed with sulfide levels that were measured by the MBB-method. While this suggest that although both measures are related to sulfide release from bound sulfide reserves, they specifically capture distinct biological phenomena with clinical relevance. Hine and colleagues have recently performed a thorough chemical characterization of the mechanisms of non-enzymatic H_2_S production in blood, demonstrating that it is catalyzed by iron and vitamin B_6_ (PLP) with cysteine serving as optimum substrate (25). Although this work gives us many clues, important questions remain about what specific components of the plasma determine and regulate H_2_S production capacity. One theory revolves around the sulfur containing amino acid homocysteine, which in itself has been long associated with cardiovascular disease risk, but its mechanism of pathology is not well-understood (32). As reported by Nakano et al. (33) and measured via the quantitative analysis of reactive sulfur species using MBB-method similar to our present study, homocysteine captures H_2_S and/or HS^−^ to form a homocysteine persulfide in cardiac tissue, which could potentially interfere with H_2_S-related cardiovascular protection (33). Thus, if surgical patients with decreased survival showed an increase in plasma homocysteine levels, then this could explain the dichotomy in H_2_S release in these patients using the two different methods (lead acetate vs. MBB). Furthermore, in the non-enzymatic production of H_2_S in plasma catalyzed by iron described previously (25) and confirmed here, PLP still acts as an important co-factor by the formation of a Schiff base and subsequent cysteine-aldimine and thiazolidine five-member ring intermediates prior to iron rapidly catalyzing the release of the sulfide. Importantly, homocysteine itself can also form a Schiff base with PLP, except it will result in a more thermodynamically stable six-member tetrahydrothiazine ring (34). The formation of the stable tetrahydrothiazine ring poses two complications for H_2_S production in circulation: the first being sequestration of valuable PLP co-factor from both enzymatic and non-enzymatic H_2_S production where cysteine serves as substrate; and the second being slow H_2_S production kinetics when iron serves as a catalyst (25), which we demonstrate here. Thus, the suppression of H_2_S release by increased circulating homocysteine may represent a mechanism explaining the robust association between homocysteine levels and cardiovascular disease.

In addition to addressing the issues brought up in the discussion, the results reported here will aid in the development of specific H_2_S assays for diagnostic purposes. Further, they will guide therapeutic interventions if these specific determinants are shown to be causal to the disease process. Despite these remaining questions, we demonstrate that the lead acetate assay represents a simple, rapid and very low-cost method which appears to capture substantial information on clinical risk in this population.

Limitations of our study include the quantification of H_2_S using the lead acetate method measures only relative differences in H_2_S between individuals and groups, and not absolute differences. Likewise, it primarily serves as a surrogate for the actual amount of H_2_S produced from available substrates in plasma. It is also important to note that mortality outcomes in this study encompassed two uniquely different patient populations, patients with carotid stenosis and patients with PAD necessitating surgical revascularization or amputation. These differences may influence disparities in mortality outcomes and future work will need to validate these findings in larger cohorts with more homogenous interventions. In addition, we previously demonstrated that dietary restriction (2, 35) or the hypothalamic-pituitary axis (36) were important regulators of endogenous H_2_S production and downstream signaling (37, 38). These factors, including circulating homocysteine levels, were not specifically assessed here, however, they do illuminate the possibilities that patients with increased survival post-surgery having an H_2_S production promoting diet and/or endocrine makeup in the days/hours leading to surgery. Nevertheless, we highlight our important results showing that both H_2_S production capacity and MBB-method-measured sulfide levels in vascular patients were significantly reduced compared to healthy subjects, indicating a potential correlation between H_2_S and the progression of cardiovascular pathology.

## Conclusion

This study shows that patients with vascular disease have significantly decreased circulating H_2_S production capacity and sulfide concentrations, compared to subjects with no clinical evidence of coronary or peripheral artery disease. In addition, the lead acetate H_2_S detection represents a simple, rapid and low-cost method to capture clinical risk in patients undergoing vascular surgery. Altogether, results provide further insights into the role of H_2_S biology in surgical patients and open an avenue for the use of H_2_S for diagnostics and therapeutics in those with dysfunction of their vascular system.

## Data Availability Statement

The original contributions presented in the study are included in the article/Supplementary Material, further inquiries can be directed to the corresponding author/s.

## Ethics Statement

The studies involving human participants were reviewed and approved by Partners Human Research Committee institutional review board. The patients/participants provided their written informed consent to participate in this study.

## Author Contributions

AL, MM, and KT: participated in the conception, design of the work and the acquisition, and analysis and interpretation of data for the work. JG, CM, PK, WK, GS, MT, SM, TD, JY, and PN: participated in the acquisition and analysis and interpretation of data for the work. CO, CH, and JM: participated in the conception, design of the work, and analysis and interpretation of data for the work. All authors contributed to the article and approved the submitted version.

## Funding

The Swiss National Science Foundation (PZ00P3-185927) to AL, the National Institutes of Health (R01HL148352) to CH, the Hungarian Thematic Excellence Program, the Hungarian National Research, Development and Innovation Office to PN (TKP2020-NKA-26, KH_126766, and K_129286), and the National Institutes of Health (1P01AG055369-01A1) to JM and SM.

## Conflict of Interest

The authors declare that the research was conducted in the absence of any commercial or financial relationships that could be construed as a potential conflict of interest.

## Publisher's Note

All claims expressed in this article are solely those of the authors and do not necessarily represent those of their affiliated organizations, or those of the publisher, the editors and the reviewers. Any product that may be evaluated in this article, or claim that may be made by its manufacturer, is not guaranteed or endorsed by the publisher.
