# Supplementary material for: Plasma Hydrogen Sulfide Is Positively Associated With Post-operative Survival in Patients Undergoing Surgical Revascularization
Source: Front Cardiovasc Med. 2021 Oct 25;8:750926. doi: 10.3389/fcvm.2021.750926 (PMC8574965; doi:10.3389/fcvm.2021.750926)
Supplement: Supplementary file 1 [file Data_Sheet_1.docx]

**SUPPLEMENTAL MATERIAL**
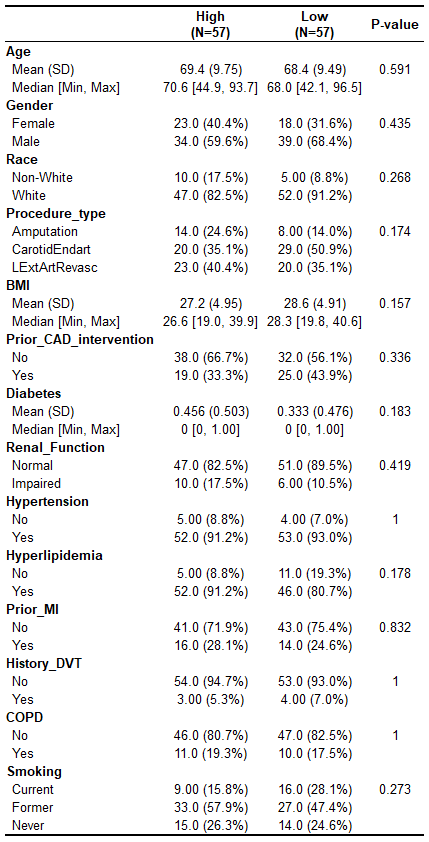


**Table S1.** Baseline Study Population Characteristics of High and Low H_2_S producers.


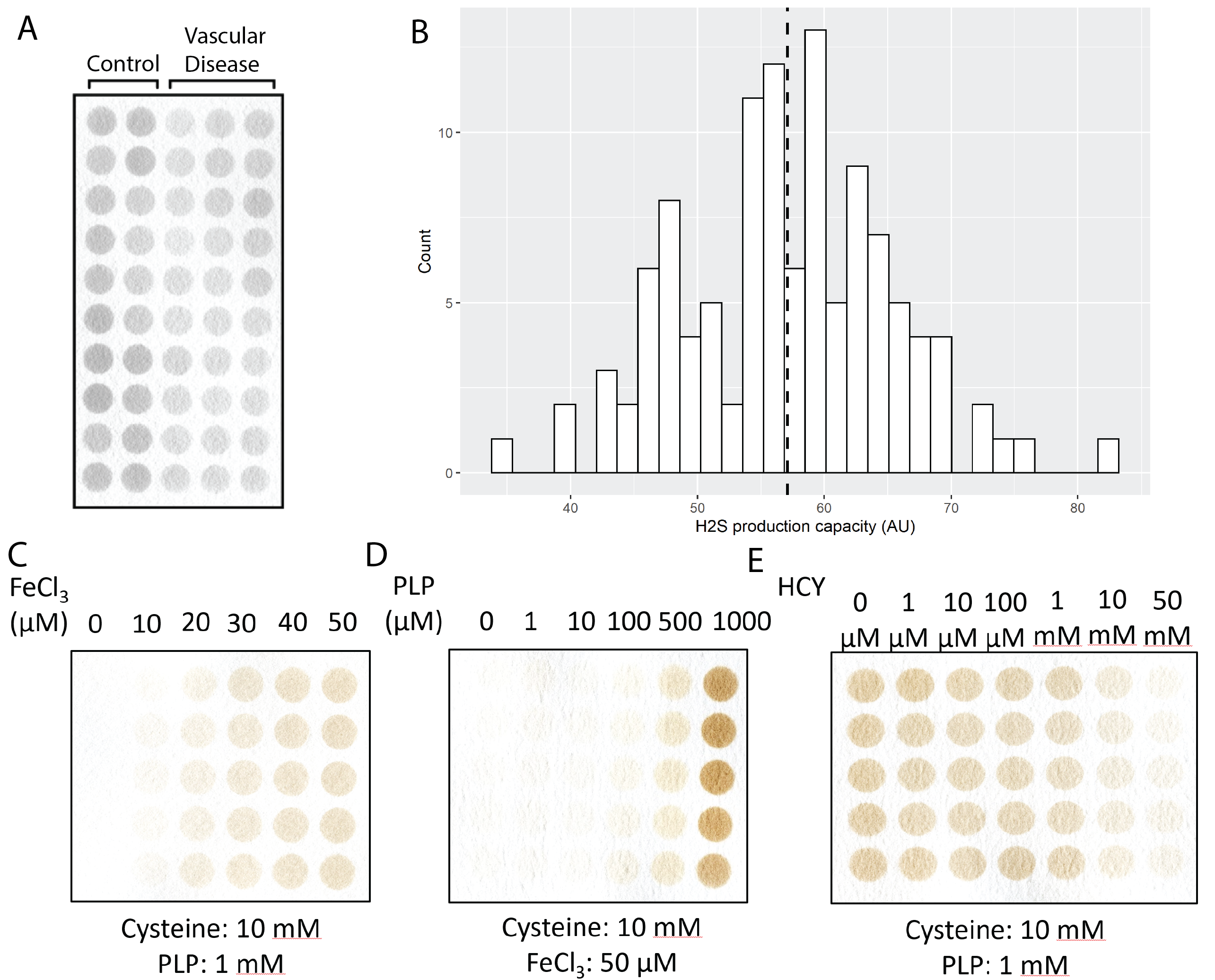


**Figure S1. Lead acetate measurements. (A)** Plasma lead acetate H_2_S release from healthy controls and vascular disease cohort. **(B)** Histogram of quantitated H_2_S release values from A. **(C)** *In vitro* H_2_S release performed using cysteine and increasing concentration of FeCl_3_, **(D)** PLP or **(E)** homocysteine (n = 5 reactions per condition). Images correspond to quantitation presented in figure 3.
